# Prognostic Value of GRACE Risk Scores in Patients With Non-ST-Elevation Myocardial Infarction With Non-obstructive Coronary Arteries

**DOI:** 10.3389/fcvm.2021.582246

**Published:** 2021-02-16

**Authors:** Guoqing Yin, Fuad A. Abdu, Lu Liu, Siling Xu, Bin Xu, Yanru Luo, Xian Lv, Rui Fan, Wenliang Che

**Affiliations:** ^1^Department of Cardiology, Shanghai Tenth People's Hospital, Tongji University School of Medicine, Shanghai, China; ^2^Department of Cardiology, Clinical Medical College of Shanghai Tenth People's Hospital, Nanjing Medical University, Shanghai, China; ^3^Department of Cardiology, Shanghai Tenth People's Hospital Chongming Branch, Shanghai, China

**Keywords:** AMI, MINOCA, non-ST-elevation, GRACE risk score, clinical outcome

## Abstract

Myocardial infarction with non-obstructive coronary arteries (MINOCA) is a special type of myocardial infarction (MI). The GRACE risk score is commonly used to predict major adverse cardiovascular events (MACE) in non-ST-elevation myocardial infarction patients, and the suitability of the GRACE risk score for prognostic stratification in patients with MINOCA remains uncertain. This study aimed to investigate whether the GRACE risk score is capable of predicting MACE in MINOCA patients with NSTE. We calculated the GRACE risk score for 340 consecutive MINOCA patients with NSTE. Patients were divided into a low-intermediate risk group (≤ 140, 48.8%) and a high risk group (>140, 51.2%) according to their GRACE risk scores. The clinical characteristics and outcomes of the patients were assessed. Patients in the high risk group tended to be older and to have more comorbidities. At the 1-year follow-up, the rate of cardiac death in the high risk group was significantly higher than that in the low-intermediate-risk group (*p* = 0.010). There was no significant difference in non-fatal MI, stroke, heart failure, or cardiovascular-related rehospitalization. The incidence of total MACE was significantly higher in patients with high GRACE risk scores than in patients with low GRACE risk scores (*p* = 0.006). ROC curve analysis showed that the GRACE risk score has moderate value in predicting MACE in NSTE-MINOCA patients. The area under the ROC curve was 0.710 (95% CI 0.625–0.796, *P* < 0.001). The GRACE risk score provides potentially valuable prognostic information on clinical outcome when applied to MINOCA patients with NSTE.

## Introduction

Myocardial infarction with non-obstructive coronary arteries (MINOCA) is a puzzling clinical entity that has been increasingly identified by coronary angiography during acute myocardial infarction (MI) and is characterized by clinical evidence of MI with normal or near-normal coronary arteries (1, 2). MINOCA can manifest as ST-elevation (STE) or non-ST-elevation (NSTE) on an electrocardiogram (ECG), and patients with NSTE are more prevalent in the MINOCA population than those with STE (1, 3). Several studies have demonstrated that MINOCA patients have a better prognosis than MI-CAD patients (3, 4); however, there are numerous reports that MINOCA has a similar prognosis to MI-CAD (5, 6) and that MINOCA patients have a higher incidence of MACE than the general population (7, 8).

Risk stratification with a specific risk score can provide an estimate of patient prognosis and optimize clinical strategies. Multiple validated risk stratification scoring systems have been established to calculate the adverse outcome risks of patients with acute coronary syndrome (ACS), such as the GRACE risk score (9–11), the TIMI score (9) and the PURSUIT score (9). Among these, the GRACE risk score is commonly used in the prediction of low and high risk of adverse outcomes due to MACE in ACS (11–13). It is the preferred risk score in clinical practice guidelines (13, 14) and considered the gold standard for the initial risk assessment of patients with suspected ACS in clinical settings. However, the suitability of the GRACE risk score for prognostic stratification in patients with MINOCA remains uncertain.

This study aimed to investigate whether the GRACE risk score is suitable to predict 1-year MACE in MINOCA patients with NSTE (NSTE-MINOCA).

## Methods

### Study Population

This was an observational and retrospective study of patients admitted to Shanghai Tenth People's Hospital and Chongming Second People's Hospital between January 2013 and April 2019 for AMI who underwent angiography during their hospitalization.

The inclusion criteria of our study were as follows: (1) meet the diagnostic criteria for MINOCA, which required meeting three criteria from the ESC guidelines (15): First, a definite diagnosis of AMI must be made (1); second, coronary angiography shows non-obstructive coronary disease, that is, no obstructive coronary disease (<50% stenosis) is found in any possible infarction-related angiography; third, no other specific alternate diagnosis for the clinical presentation; (2) ECG with NSTE present; and (3) alive at the time of hospital presentation. The exclusion criteria were as follows: (1) age <18; (2) ECG with STE; (3) types 3–5 myocardial infarction; (4) diagnosis of MINOCA was consistent with the clinical recommendations from the 4th Universal Definition of Myocardial Infarction (UDMI) published in 2018 (1), and patients presenting with a classic myocarditis presentation at enrollment, pulmonary embolism, and Takotsubo syndrome were excluded. Basic information (such as age, sex, and body mass index) and past medical history (such as hypertension, diabetes mellitus, dyslipidemia, smoking status, peripheral vascular disease, chronic heart failure, stroke, and chronic obstructive pulmonary disease) were recorded in detail. Fasting blood within 24 h of admittance was collected for assessing blood cardiac troponin-T (cTnT), creatine kinase-MB (CKMB), myoglobin, and N-terminal pro-brain natriuretic peptide (NT proBNP). In addition, medications for all patients were derived from their medical records.

To determine the final cause of MINOCA, left ventricular angiography and echocardiography were performed to assess wall motion, and intravascular ultrasonography (IVUS) or optical coherence tomography (OCT) was only used to identify atherosclerotic plaque disruption or plaque erosion in selected patients due to its poor cost-effectiveness and insurance unfeasibility.

The study complied with the Declaration of Helsinki and was approved by the hospital's ethical review board (Shanghai Tenth People's Hospital, Tongji University, Shanghai, China). Informed consent was signed by each participant in this study.

### The GRACE Risk Score Assessment

The GRACE risk scores were calculated on admission from the following eight clinical parameters: age; heart rate; systolic blood pressure (SBP); serum creatinine; Killip classification; cardiac arrest; ST-segment deviation on ECG; and elevated cardiac enzyme. All eight parameters were in line with the GRACE definitions. For each individual participant, the GRACE risk score was calculated, and the participants were divided into two groups according to their GRACE score: the low-intermediate risk (≤ 140) group and the high risk (>140) group.

### Follow-Up

Follow-ups were conducted by experienced cardiologists in Shanghai Tenth People's Hospital and Chongming Second People's Hospital for 1 year. Patients were monitored *via* telephone or clinic visits. We asked our patients to visit the hospital to evaluate their adherence and persistence status. Questions were asked regarding rehospitalization for heart disease, the incidence of complications and the utilization of medicine during the 1 year after hospital discharge. Follow-up data were available for 307 (90.1%) patients. The follow-up endpoint of the study was the combined occurrence of MACE, defined as cardiac death, non-fatal MI, stroke, heart failure, and cardiovascular-related rehospitalization. Cardiac death was defined as death due to arrhythmia, heart failure, endocarditis, or sudden death without another explanation available. MI was defined as characteristic ECG changes or an increase in cardiac troponin I (>0.1 ng/ml) with typical ischemic symptoms (1). Stroke was defined as an ischemic cerebral infarction caused by any major intracranial artery occlusion verified by imaging (16). Heart failure was diagnosed according to the current guidelines (17). Cardiovascular rehospitalization was rehospitalization for cardiac causes, such as angina, or other cardiovascular diseases with positive cardiac biomarkers.

### Statistical Analysis

Continuous variables with a normal distribution are presented as the mean ± standard deviation, continuous variables with a skewed distribution are presented as the median, and categorical variables are given as percentages. Student's *t*-test was used to compare significant differences in consecutive variables between groups. Pearson's chi-squared test or Fisher's exact test was used to determine significant differences between categorical variables. Goodman and Kruskal γ statistical method was used to calculate the association between GRACE risk score and severity of coronary artery lesions Logistic regression models were used to derive adjusted ORs for MACE to quantify the relative risk of outcomes in the low-intermediate risk group and high risk group patients. Covariates in the models were sex, hypertension, diabetes mellitus, smoking, heart failure, and stroke. Receiver operating characteristic (ROC) analysis was used to quantify the ability of the GRACE risk score to estimate MACE in NSTE-MINOCA patients. The definition of the Youden index is sensitivity plus specificity −1, and the highest Youden index corresponds to the optimal cut-off value. A two-tailed *p*-value below 0.05 was considered significant. No adjustment was made for multiplicity. Statistical analyses were performed using Statistical Package for Social Sciences (SPSS) v.22 software for Windows 10.

## Results

### Baseline Characteristics of the Patients

The subjects of this study were 5,863 consecutive AMI patients, of which 340 (5.8%) patients met the diagnostic criteria for NSTE-MINOCA. According to the GRACE risk score results, 166 (48.8%) patients were included in the low-intermediate risk group, while 174 (51.2%) were included in the high risk group. The number of patients who underwent IVUS and OCT was 69 and 34, respectively. Patient characteristics based on the GRACE risk score are listed in Table 1.

**Table 1:** Baseline characteristics of NSTE-MINOCA patients according to GRACE risk score.

	**Low-intermediate**	**High risk**	***P*-value**
	**risk**		
	**(*n* = 166)**	**(*n* = 174)**	
**Demographics**			
Age (years)	54.2 ± 10.7	70.8 ± 10.2	<0.001
Sex (females), *n* (%)	68 (41.0)	94 (54.0)	0.016^a^
BMI (kg/m^2^)	24.7 ± 3.7	24.2 ± 3.9	0.227
**Medical history and clinical measures**			
Hypertension, *n* (%)	70 (42.2)	92 (52.9)	0.048^a^
Diabetes mellitus, *n* (%)	10 (6.0)	33 (19.0)	<0.001^a^
Dyslipidemia, *n* (%)	26 (15.7)	21 (12.1)	0.337^a^
Smoking, *n* (%)	79 (47.6)	80 (46.0)	0.766^a^
Peripheral vascular disease, *n* (%)	10 (6.0)	21 (12.1)	0.053^a^
Chronic heart failure, *n* (%)	2 (1.2)	7 (4.0)	0.175^b^
Stroke, *n* (%)	10 (6.0)	31 (17.8)	0.001^a^
COPD, *n* (%)	1 (0.6)	4 (2.3)	0.372^b^
Mean heart rate (b.p.m.)	79.8 ± 16.7	82.8 ± 19.8	0.133
Systolic blood pressure (mmHg)	148.9 ± 23.1	134.7 ± 21.7	<0.001
Cardiac arrest (pre-hospital), *n* (%)	0	1 (0.6)	1.000^b^
Initial creatinine (mmol/L)	72.3 ± 21.2	92.3 ± 51.9	<0.001
Initial cardiac biomarker positive,	130 (78.3)	162 (93.1)	<0.001^a^
*n* (%)			
ST-deviation on admission, *n* (%)	75 (45.2)	57 (32.8)	0.019^a^
LVEF (%)	57.4 ± 10.8	52.1 ± 12.5	<0.001
**Angiographic data**			0.003
Normal-appearing vessels, *n* (%)	86 (51.8)	73 (42.0)	
Vessel with any stenosis			
1-vessel disease, *n* (%)	38 (22.9)	48 (27.6)	
2-vessel disease, *n* (%)	37 (22.3)	28 (16.1)	
3-vessel disease, *n* (%)	5 (3.0)	25 (14.4)	

*BMI, body mass index; COPD, chronic obstructive pulmonary disease; LVEF, left ventricular ejection fraction*.

a *Pearson's chi-squared test*.

b *Fisher's exact test*.

Patients with high risk scores were more likely to be older (54.2 ± 10.7 vs. 70.8 ± 10.2, *p* < 0.001) and female (41.0 vs. 54%, *p* = 0.016) and had a higher prevalence of comorbidities such as diabetes mellitus, smoking, and stroke. Furthermore, compared to patients with a high risk score, patients with a low-intermediate risk had a higher SBP (148.9 ± 23.1 vs. 134.7 ± 21.7, *p* < 0.001), whereas their initial creatinine was lower (72.3 ± 21.2 vs. 92.3 ± 51.9, *p* < 0.001). The proportion of patients with initial cardiac biomarker positivity was greater in the high risk group (78.6 vs. 93.4%, *p* = 0.001), while ST deviation on ECG on admission was more common in the low-intermediate risk group (45.2 vs. 32.8%, *p* = 0.019). Echocardiography revealed that the left ventricular ejection fraction (LVEF) in the high risk group was significantly lower than that in the low-intermediate risk group (57.4 ± 10.8 vs. 52.1 ± 12.5, *p* < 0.001). Angiography data showed a weak positive association between GRACE risk score and coronary artery lesions (γ = 0.185, *p* = 0.03).

### Medications

Discharge medications and 1-year follow-up medications are shown in Table 2. Patients in the high risk group were more likely to be discharged on clopidogrel, and there were no significant differences in the use of other medication treatments. At the 1-year follow-up, the proportion of patients using statins and clopidogrel was slightly higher in patients with higher GRACE risk scores.

**Table 2:** Medication treatment at discharge and 1-year follow-up.

**Medication**	**At discharge**		***P*-value**	**1-year follow-up**		***P*-value**
	**Low-intermediate risk**	**High risk**		**Low-intermediate risk**	**High risk**	
	**(*n* = 166)**	**(*n* = 174)**		**(*n* = 147)**	**(*n* = 160)**	
Statins, *n* (%)	122 (73.5)	133 (76.4)	0.531	60 (40.8)	88 (55.0)	0.013
Aspirin, *n* (%)	101 (60.8)	96 (55.2)	0.290	58 (39.5)	64 (40.0)	0.922
Clopidogrel, *n* (%)	57 (34.3)	86 (49.4)	0.005	26 (17.7)	45 (28.1)	0.030
ACEI/ARB, *n* (%)	68 (41.0)	78 (44.8)	0.472	44 (29.9)	60 (37.5)	0.162
β-BLOCK, *n* (%)	83 (50.0)	88 (50.6)	0.916	51 (34.7)	65 (40.6)	0.284
CCB, *n* (%)	62 (37.3)	58 (33.3)	0.439	43 (29.3)	39 (24.4)	0.335

*ACEI, Angiotensin-converting enzyme inhibitors; ARB, Angiotensin receptor blocker; CCB, Calcium channel blocker*.

### Clinical Outcomes During the 1-Year Follow-Up

The follow-up data were available for 307 patients (90.3%), 147 of which were in the low-intermediate risk group (88.6% follow-up) and 160 of which were in the high risk group (92.0% follow-up). At the 1-year follow-up, a total of 50 cases of MACE occurred. In the low-intermediate risk group, 15 MACE occurred (2 cardiovascular deaths, 1 non-fatal MI, 1 stroke, 0 heart failure, and 11 cardiovascular-related rehospitalizations). In the high risk group, 35 MACE occurred (12 cardiovascular deaths, 0 non-fatal MI, 2 stroke, 3 heart failure, and 18 cardiovascular-related rehospitalizations) (Table 3). The rate of cardiac death in the high risk group was significantly higher than that in the low-intermediate-risk group (*p* = 0.010). The incidence of total MACE was significantly higher in patients with high GRACE risk scores than in patients with low GRACE risk scores (*p* = 0.006). Among all NSTE-MINOCA patients, the low-intermediate risk group patients had an unadjusted OR of 0.402 (95% CI 0.209–0.774, *p* = 0.006) for MACE compared with the high risk group patients. After adjusting for sex, hypertension, diabetes, smoking, heart failure, and stroke, the adjusted OR for low-intermediate risk group patients was 0.431 (95% CI 0.218–0.856, *P* = 0.016) compared with the high risk group patients (Table 4).

**Table 3:** Major adverse events based on GRACE risk score.

	**Low-intermediate**	**High risk**	***P*-value**
	**risk group**	**group**	
	**(*n* = 147)**	**(*n* = 160)**	
**MACE**	15 (10.2)	35 (21.9)	0.006^a^
Cardiac death, *n* (%)	2 (1.4)	12 (7.5)	0.010^a^
Non-fatal MI, *n* (%)	1 (0.7)	0	0.479^b^
Stroke, *n* (%)	1 (0.7)	2 (1.3)	1.000^b^
Heart failure, *n* (%)	0	3 (1.9)	0.249^b^
Cardiovascular-related	11 (7.5)	18 (11.3)	0.260^a^
rehospitalization, *n* (%)			

MACE, major adverse cardiovascular events; MI, myocardial infarction.

a Pearson's chi-squared test.

b *Fisher's exact test*.

**Table 4:** Risk of MACE in low-intermediate risk group compared with high risk group.

	**Unadjusted**			**Adjusted for A**		
	**OR**	**95% CI**	***P*-value**	**OR**	**95% CI**	***P*-value**
Low-intermediate risk group	0.402	0.209–0.774	0.006	0.431	0.218–0.856	0.016

*A = sex, hypertension, diabetes mellitus, smoking, heart failure, and stroke; OR, odds ratio; CI, confidence interval*.

### Predictive Performance of the GRACE Risk Score

**Figure 1:**
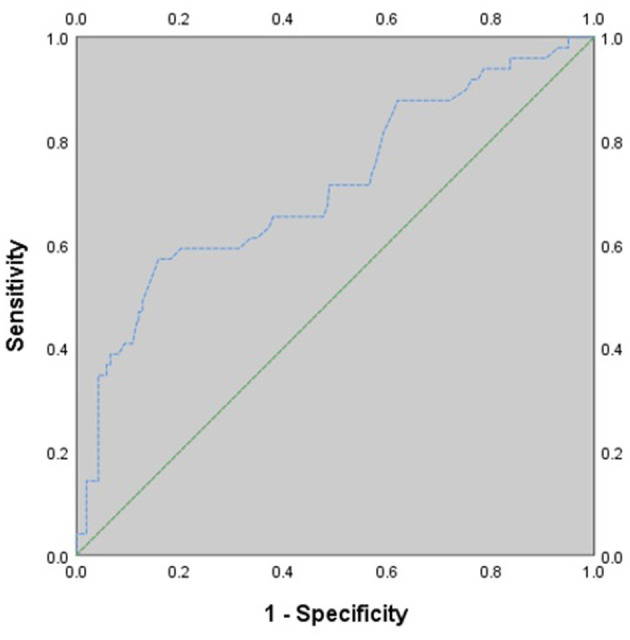
ROC curve for 1-year MACE. AUC, area under the curve; CI, confidence interval.

Figure 1 shows the ROC curve for the GRACE risk score in predicting 1-year MACE in NSTE-MINOCA patients. ROC curve analysis demonstrated that the GRACE risk score has moderate value in predicting MACE in NSTE-MINOCA patients [area under the curve (AUC) = 0.710, 95% CI 0.625–0.796, *P* < 0.001]. The highest Youden index was 0.413, and the corresponding optimal cut-off value of the GRACE score was 159. The sensitivity and specificity were 57.1, 84.1%, and positive and negative predictive values were 34.1% (95% CI 28.2–40.0%) and 91.8% (95% CI 87.9–95.7%) at the threshold.

## Discussion

The objective of this study was to investigate whether the GRACE risk score is capable of predicting 1-year MACE in MINOCA patients with NSTE. Our major finding was that the incidence of total MACE was significantly higher in the high risk group than in the low-intermediate risk group based on the GRACE risk score. ROC analysis demonstrated that the GRACE risk score had a moderate discrimination ability to stratify NSTE-MINOCA patients by their risk of MACE. The data showed that the GRACE risk score has prognostic value in patients with NSTE-MINOCA.

In recent years, the prognosis of MINOCA has gained attention because MINOCA has been recognized as having a prevalence of 5–15% (2, 3, 8, 18–20). A systematic review (3) reported that patients with MINOCA had a lower all-cause mortality rate than those with MI-CAD, and the 1-year all-cause mortality rate of MINOCA patients was 4.7%. A meta-analysis (21) revealed that all cardiovascular outcome event rates (MACE, all deaths, cardiac death, MI, and all deaths plus MI) in non-obstructive CAD were significantly lower than those in obstructive CAD. A recent study (19) on Chinese MINOCA patients indicated that the occurrence of MACE (cardiovascular deaths, non-fatal MI, strokes, heart failures, and cardiovascular-related rehospitalizations) was lower in the MINOCA group than in the MI-CAD group at the 1-year follow-up. Although compared with that of MI-CAD, the prognosis of MINOCA is slightly better, it is not a benign disease (2, 3, 18, 22, 23). Some studies (5, 24) indicated that patients with MINOCA had clinical outcomes that were similar to those of MI-CAD patients. Another study (25) confirmed an unfavorable prognosis in elderly patients with MINOCA undergoing coronary angiography, with one in five patients with MINOCA suffering a major adverse event at the 1-year follow-up. In addition, two studies (7, 8) that compared MINOCA patients with the general population showed that MINOCA patients have a higher incidence of MACE.

Risk stratification tools can help objectify the clinical triage process and quantify the probability of serious morbidity and mortality. When applied appropriately, risk scores can be useful in aiding cardiologists in developing optimal treatment strategies. To our knowledge, there is currently no score for predicting the prognosis of MINOCA. MINOCA is a type of MI, but it is unclear whether the risk stratification tools for MI patients are also applicable to MINOCA patients. The GRACE risk score has some desirable features for ACS patients because it is easy to calculate and widely used, and it relies on clinical data that are easily obtainable in hospitals. However, whether the GRACE risk score is suitable for MINOCA is uncertain.

The GRACE risk score, which was developed by Granger et al. (11) in 2002, was established using a large multinational cohort to predict in-hospital mortality in ACS patients. Numerous studies (12, 26, 27) have verified the accuracy of the GRACE risk score in all types of ACS patients. It was recommended by the guidelines (13, 14) developed by both the European Society of Cardiology and the American College of Cardiology/American Heart Association for initial risk stratification in NSTE-ACS patients. Two studies (28, 29) have shown that the GRACE risk score also has predictive value for long-term prognosis in patients with ACS, and another study (30) has shown the validity of the GRACE risk score in the long-term prognosis of elderly AMI patients. A recent study (31) validated the accuracy of the GRACE score for risk stratification in contemporary management of NSTE-ACS. Another study (32) demonstrated that the GRACE risk score has clinical applicability in ACS patients with diabetes. In addition, Sergio et al. (33) found that the GRACE risk score is a useful tool for predicting contrast-induced nephropathy in patients with MI and normal renal function. Previous studies (28, 31) have stratified NSTE-ACS patients based on their GRACE risk scores, and patients with higher scores tend to have worse clinical outcomes. Considering that the GRACE risk score stratifies NSTE-ACS patients and that stratification levels correlate with clinical outcomes, we hypothesized that the GRACE risk score may be useful for risk stratification and prognosis prediction in NSTE-MINOCA patients.

Previous studies on GRACE risk scores in ACS patients usually divided patients into a high risk group (>140), an intermediate-risk group (109–139) and a low-risk group (<109) based on their GRACE risk scores. In the present study, as only 33 patients were identified for the low-risk group, their data were merged with the data of those in the intermediate-risk group for further statistical analysis, and all patients were divided into two main groups according to the score obtained: the high risk group (≤ 140) and the low-intermediate-risk group (>140). The data showed that the rate of cardiac death in the high risk group was significantly higher than that in the low-intermediate-risk group, although there was no significant difference in the rates of non-fatal MI, stroke, heart failure, and cardiovascular-related rehospitalization. Several reasons might account for this result. First, some potential risk factors (such as smoking status, diabetes mellitus, and hypertension) for MI, stroke, and heart failure were not included in the GRACE risk score. In addition, the small sample size and short follow-up period might be other reasons. The rate of total MACE was significantly higher in the high risk group, which showed that the GRACE risk score has clinical value in NSTE-MINOCA patients. In addition, patients in the low-intermediate group had an unadjusted OR of 0.402 for MACE when compared with the high risk group patients. Similarly, after adjusting for related cardiovascular disease risk factors, we found that the adjusted OR remained unchanged in the low-intermediate group. This result demonstrated that the low-intermediate group might be independently associated with a lower risk of MACE, which indicates that the GRACE risk score could have clinical ability to stratify NSTE-MINOCA patients and their risk of MACE within 1 year. Furthermore, we used ROC curve analysis to determine the predictive value of the GRACE risk score in NSTE-MINOCA patients, and the data showed that the GRACE risk score had a moderate ability to categorize NSTE-MINOCA patients by their risk of MACE. This indicated that the GRACE risk score may be an acceptable method for risk stratification and prognosis prediction in patients presenting with NSTE-MINOCA, which needs to be confirmed through large sample size, multicenter, prospective studies.

Several limitations should be considered in this study. First, the study population was relatively small, and the numbers are not powered enough to make final conclusions. Second, our main objective was to study NSTE-MINOCA patients. As such, our results cannot be generalized to all MINOCA patients. Our findings need further studies with a larger number of participants to be confirmed. Our findings should be regarded as preliminary, indicative of the need for new risk stratification scores for MINOCA patients.

## Conclusion

This is the first study on GRACE risk scores in patients with NSTE-MINOCA, and it showed that GRACE risk scores provide potentially valuable prognostic information on clinical outcome when applied to NSTE-MINOCA patients.

## Data Availability Statement

The raw data supporting the conclusions of this article will be made available by the authors, without undue reservation.

## Ethics Statement

The studies involving human participants were reviewed and approved by Shanghai Tenth People's Hospital, Tongji University, Shanghai, China. The patients/participants provided their written informed consent to participate in this study.

## Author Contributions

GY, FA, and WC designed the study, drafted the manuscript, and revised it critically for important intellectual content. LL, BX, FA, and GY collected the data. BX, YL, SX, XL, and RF were involved in data cleaning, follow-up, and verification. FA and WC analyzed the data. WC approved the final version of the manuscript. All authors contributed to manuscript revision, read, and approved the submitted version.

## Conflict of Interest

The authors declare that the research was conducted in the absence of any commercial or financial relationships that could be construed as a potential conflict of interest.
